# Abortion knowledge, attitudes and reproductive autonomy among university students in Austria: a cross-sectional study

**DOI:** 10.3389/frph.2026.1820413

**Published:** 2026-05-22

**Authors:** Bettina Böttcher, David Riedl, Elisabeth Reiser, Magdalena Flatscher-Thoeni, Bettina Toth

**Affiliations:** 1 Department of Gynecological Endocrinology and Reproductive Medicine, Medical University of Innsbruck, Innsbruck, Austria; 2 Department of Psychiatry, Psychotherapy, Psychosomatics and Medical Psychology, University Hospital of Psychiatry II, Medical University of Innsbruck, Innsbruck, Austria; 3 Ludwig Boltzmann Institute for Rehabilitation Research, Vienna, Austria; 4 Department of Public Health, Health Services Research and Health Technology Assessment, UMIT TIROL – University for Health Sciences and Technology, Innsbruck, Austria

**Keywords:** abortion, autonomy, beginning of life, coercion, reproduction

## Abstract

**Background:**

Abortion laws are widely debated, yet little is known about how abortion knowledge relates to reproductive autonomy among university students. This study assessed Austrian students' abortion knowledge, attitudes, and reproductive autonomy, and examined factors associated with liberal vs. conservative positions.

**Methods:**

A cross-sectional online survey was conducted in 2023 among students in Tyrol, Austria (*n* = 750). The questionnaire included sociodemographics, prior experiences, abortion knowledge (law, access, cost), attitudes, and the 14-item Reproductive Autonomy Scale (RAS). Analyses included ANOVA, Kruskal–Wallis, Mann–Whitney U, *χ*² tests, and Spearman correlations (*α* = 0.05).

**Results:**

Participants were 622 women and 128 men (median age 23 years). Thirty-three (4.4%) had children; 67.5% desired children; 39.9% had used emergency contraception; 25 women had undergone abortion. Only 49.7% knew abortion is legally regulated in Austria, with higher knowledge among medical students (62.2%) than other health (44.7%) or non-health students (41.6%; *p* < 0.001). Women more often identified the first-trimester regulation (74.6% vs. 60.9%; *p* = 0.012). Knowledge of providers and costs was generally poor, though slightly better among medical students. A social taboo was more frequently perceived by women (81.6% vs. 65.1%; *p* < 0.001). When asked when life begins, 36.1% selected “heartbeat” and 27.0% “viability.” Higher RAS scores correlated with more liberal abortion attitudes (*r* = .16–.17; *p* ≤ 0.001).

**Conclusions:**

In this sample of Austrian students, strong support for reproductive autonomy but limited knowledge of abortion law, access, and costs is shown. Greater reproductive autonomy aligns with more liberal attitudes. Findings support integrating structured abortion education, improving information on access, and reducing stigma.

## Introduction

Globally, abortion legislation is undergoing significant and often divergent transformations. While some countries have recently enacted more restrictive regulations, a pronounced international trend toward liberalization and decriminalization is evident (1, 2). For instance, the Maputo Protocol has played a pivotal role in advancing reproductive rights across several African nations (3), and recent legal reforms in Latin America and parts of Europe have further expanded access to abortion services (4).

These contrasting developments have intensified societal and ethical debates. Questions surrounding abortion and the definition of the beginning of human life are at the forefront of public discourse. Demands for decriminalization resonate strongly, both in Austria and globally, reflecting ongoing tensions among legal, moral, and public health perspectives.

When examining societal perspectives on abortion, one can broadly classify them into three categories: conservative, moderate, and liberal. The conservative viewpoint asserts that human life begins at the moment of fertilization, granting it the same rights to life as the pregnant individual. Within this framework, the embryo or fetus is viewed as having an inherent claim to protection; thus, abortion is generally rejected, and pregnancy conflicts are typically resolved in favor of the embryo. In situations involving medical indications for termination, opinions remain divided, as both the pregnant individual and the fetus are seen as having equal rights and a need for protection (5).

Moderate approaches acknowledge specific exceptions that may justify abortion. These views frequently incorporate gestational limits beyond which abortion is no longer considered ethically acceptable (5, 6). Rather than equating fertilization with the inception of life, they seek to balance the interests of the pregnant individual with those of the prenatal child.

In contrast, liberal perspectives advocate for broad access to abortion, some interpretations even permitting it at any stage of pregnancy, and generally prioritize the rights and autonomy of the pregnant individual. Within this framework, two main argumentative lines emerge in the literature: one emphasizes the right to bodily self-determination, asserting that this right outweighs any potential claims of the fetus, regardless of its moral status (7). The second focuses on the moral status of the fetus, arguing that personhood and the corresponding right to life are acquired only at a later stage of development, often defined by the onset of sentience or, in more radical views, at birth (5, 8). Thus, while specific nuances exist, liberal orientations emphasize the primacy of reproductive autonomy.

Recent research has illuminated how young adults perceive abortion across different cultural and national contexts. A qualitative study conducted in Croatia explored the views of young women aged 18–35 through semi-structured interviews (9). The findings reveal that cultural, religious, and familial influences play a significant role in shaping attitudes toward abortion, with many participants navigating tensions between personal beliefs and societal expectations, yet affirming a woman's right to choose.

In a cross-national quantitative study across diverse nations, researchers examined the multidimensional nature of abortion attitudes (10). Rather than a simple pro-life vs. pro-choice dichotomy, the study identified clusters of legal attitudes, including variations in acceptance based on specific indications (e.g., medical, social, or criminological) and gestational limits.

Furthermore, the European Values Survey (11) provides a large comparative dataset that sheds light on generational differences in abortion attitudes across Europe. Results indicate that a strong ethnonational identity correlates with a conservative view of abortion, a pattern that is particularly pronounced among non-religious individuals. Younger adults tend to adopt more liberal positions compared to older cohorts.

Moreover, a comparison of university students' attitudes toward both abortion and euthanasia in three European countries (12) found that field of study and religiosity are stronger predictors of abortion attitudes than gender or age. Theology students exhibited more conservative views, while those in medicine and social sciences were more supportive of abortion rights, underscoring the role of disciplinary context in shaping young adults’ ethical viewpoints.

Together, these studies underscore that while young adults across Europe tend to express more supportive views on abortion compared to older populations, their attitudes remain influenced by cultural, religious, and educational contexts. To broaden the knowledge in this field and address the limited empirical evidence from Austria, the objective of the present prospective cross-sectional study is to analyze attitudes and knowledge about abortion among students in Austria, as well as possible correlations with reproductive autonomy.

## Methods

### Participants

In this prospective cross-sectional study 937 students took part from October 2023 to November 2023 (Supplementary Table S1). A questionnaire created using the online survey platform SurveyMonkey was sent to heads of bachelor and master degree programmes in Tyrol, Austria with the request to forward the survey to the students. Students were also invited to take part in the study via social media, e.g., students associations. Participants accessed the questionnaire via a web link or a QR code. The total number of students only reflects the number of students who received access to the survey link not representing a response rate. Only students based in Tyrol were able to participate in the study, based on self- report. After providing informed consent, subjects completed a comprehensive questionnaire including their field of study and specifying their (desire on further) knowledge on abortion as well as reproductive autonomy as detailed below. No quotas or steering mechanisms were applied during recruitment. Diverse persons or non- defining themselves as male or female were excluded as this were 6 persons in our cohort and statistical analysis compared to female or male participants is not precise. The institutional ethics committee of the Medical University of Innsbruck approved the study (1012/2022).

### Questionnaire

The participants received a questionnaire on demographic data including age, religious belief, study type, monthly salary as well as personal questions about abortion, pregnancies, contraceptive methods and the use of morning- after-pill followed by specific theoretical questions on abortion like current knowledge of the as well as further desire on knowledge. It consisted of 43 questions ranging from the legal framework, sources of information, education, cost and the time of beginning of human life as and specific statements to consent to or not.

Moreover, all participants- independent from gender- were asked to complete the Reproductive Autonomy Scale (RAS) comprising 14 questions divided into three subscales. The *Freedom from Coercion* subscale includes five questions that assess potential coercion by a partner regarding contraception, contraceptive methods. The *Communication* subscale also consists of five questions, focusing on communication with a partner about contraception, sex, and pregnancy. Both of these subscales use a four-point Likert scale for responses, ranging from 0 (strongly disagree) to 3 (strongly agree). The third subscale, *Decision-Making*, contains four questions, each with three possible response options (0–2). These items address who holds decision-making power in the relationship concerning reproductive matters. All subscales are scored such that higher scores indicated greater reproductive autonomy. The questionnaire was initially designed for use with girls and women (14) and has been approved in several countries like Brazil, UK, and Ghana. The use in German language was validated and tested in a population of Austrian students (15) However, it was recommended to use the questionnaire in female participants only for the time being (14).

### Statistical analyses

Descriptive statistics are presented for the whole sample of participants who completed key sociodemographic data (nationality, age, religion, relationship status and employment status) and the RAS. Participants who only provided informed consent and no more data were not included in the analysis. The dataset contained a negligible proportion of missing values (below 1%), which did not materially affect the analysis. Therefore, no imputation methods or dummy variables were employed. Due to the low number of gender diverse individuals all subsequent analyses were only conducted for male and female participants. Analysis of variance (ANOVA) was performed for normally distributed raw data, which was presented as mean ± standard deviation (s.d.). For non-normal data distribution, the differences between the individual parameters of the groups were analyzed using the Kruskal–Wallis or Mann–Whitney *U*-test and presented as median (interquartile range [IQR]). The Spearman's rank correlation analysis was used to identify correlations between different parameters as data was not normally distributed. Association between dichotomous variables was analyzed with the chi square test. Given the exploratory nature of the study and the number of statistical tests performed, no formal correction for multiple comparisons was applied. Results should therefore be interpreted with caution. A significance level of *α* = 0.05 was selected for all statistical evaluations. The statistical analysis was conducted using IBM SPSS Statistics for Windows, version 26.0 (IBM Corp., Armonk, NY, USA).

## Results

Of 750 study participants, 622 were female and 128 male with a median age of 23.0 (21.0−25.0) years. Diverse persons or non- defining themselves as male or female were excluded as this were 6 persons in our cohort. Basic characteristics are depicted in Table 1. 33 (4.4%) participants already have children, 506 (67.5%) do want to have children in the future compared to 66 (8.8%) without any desire to get pregnant. 299 (39.9%) participants already reported that either themselves or the partner already took the morning-after pill in the past. 25 (3.3%) women already underwent an abortion.

**Table 1:** Basic demographic characteristics of the study cohort.

Characteristic	Total (*n* = 750)	Female (622)	Male (128)
Age, years (median, IQR)	23.0 (21.0–25.0)	23.0 (21.0–25.0)	23.0 (22.0–25.0)
Origin			
Rural area	412 (54.5%)	346 (55.6%)	62 (48.4%)
City	344 (45.5%)	278 (44.4%)	66 (51.6%)
Confession			
Christian	537 (71.8%)	446 (72.3%)	89 (69.5%)
No confession	148 (19.8%)	115 (18.6%)	32 (25.0%)
Other	63 (8.4%)	61 (9.1%)	7 (5.5%)
Type of studies			
Medicine	267 (35.6%)	193 (31.6%)	74 (57.8%)
Other health profession	190 (25.3%)	196 (31.5%)	10 (7.8%)
No health profession	293 (39.1%)	233 (38.1%)	44 (34.4%)
Monthly salary in Euro			
<500	180 (23.9%)	152 (24.5%)	27 (21.1%)
500–1,000	346 (45.9%)	291 (46.9%)	52 (40.6%)
>1,000	228 (30.2%)	177 (28.5%)	49 (38.3%)
Sexual Orientation			
Heterosexual	612 (81.6%)	498 (80.3%)	112 (87.5%)
Homosexual	43 (5.7%)	35 (5.6%)	8 (6.3%)
Bisexual	74 (9.9%)	67 (10.8%)	7 (5.5%)
Other	25 (3.3%)	22 (3.5%)	1 (0.7%)

### Current knowledge

Half of the students (49.7%) independent from gender know that there is a law in Austria that regulates abortion. Knowledge was highest among medical students compared to other health science and no health science (62.2% vs. 44.7% vs. 41.6%; *p* = <0.001). When it comes to the non-punished period of the first 3 months of pregnancy, female participants were better informed than male participants (74.6% vs. 60.9%, *p* = 0.012). Knowledge about facilities that perform abortions and how high the costs are was poor, irrespective of gender: 55.6% and 45.2% of total participants did not know, respectively. Only medical students had significantly better knowledge (*p* = 0.016 and *p* = 0.049). Female students in particular had the impression that abortion is a socially taboo subject compared to male students (81.6% vs. 65.1%; *p* = <0.001).

### Desire for knowledge about abortion

Furthermore, the current knowledge and desire for knowledge about abortion was surveyed. There were statistically significant differences between male and female participants (Table 2). Female participants feel less sufficiently informed about the possibility of abortion, know better where to obtain reliable information on this topic, state information as very important and wish for a compulsory abortion course in the medical curriculum.

**Table 2:** Desire for knowledge about abortion dependent on gender.

Question	Female (622)	Male (128)	*P*-value
Do you feel that you are sufficiently informed about the possibility of an abortion? 4			0.009
Yes	185 (29.7%)	52 (40.6%)	
No	435 (69.9%)	74 (57.8%)	
Do you know where you can get reliable information on this topic?			0.011
Yes	304 (48.9%)	81 (63.3%)	
No	317 (51.0%)	47 (36.7%)	
Do you think it is important to be informed about the possibilities and information here?			<0.001
Very important	477 (77.2%)	78 (60.9%)	
Important	126 (20.4)	39 (30.5%)	
Rather not important	5 (0.8)	5 (3.9%)	
Not important	10 (1.6%)	6 (4.7%)	
Should there be a compulsory course on abortion in the human medicine degree programme?			<0.001
Yes	583 (93.7)	99 (77.3%)	
no	22 (3.5)	12 (9.4%)	
I do not care	16 (2.6%)	17 (13.3%)	

When the participants were divided into groups according to their field of study, medical students and students of other health sciences felt better informed than those from other fields of study (*p* = 0.01) as depicted in Table 3.

**Table 3:** Desire for knowledge about abortion dependent on type of studies.

Characteristic	Medical studies (267)	other health sciences (190)	No health science (293)	*P*-value
Do you feel that you are sufficiently informed about the possibility of an abortion?				0.010
Yes	101 (37.8%)	63 (33.2%)	73 (24.9%)	
No	166 (62.2%)	126 (66.3%)	217 (66.3%)	
Do you know where you can get reliable information on this topic?				0.209
Yes	150 (56.2%)	95 (50.0)	140 (47.8%)	
No	117 (43.8%)	95 (50.0)	152 (51.9%)	
Do you think it is important to be informed about the possibilities and information here?				0.018
Very important	211 (79.6%)	140 (73.7%)	204 (70.1%)	
Important	45 (17.0%)	49 (25.8%)	71 (24.4%)	
Rather not important	3 (1.1%)	0	7 (2.4%)	
Not important	6 (2.3%)	1 (0.5%)	9 (3.1%)	
Should there be a compulsory course on abortion in the human medicine degree programme?				0.626
Yes	240 (89.9%)	178 (93.7%)	264 (90.1%)	
no	14 (5.2%)	5 (2.6%)	15 (5.1%)	
I do not care	12 (4.5%)	7 (3.7%)	14 (4.8%)	

When being asked about when life begins, most participants stated with the presence of a heartbeat (36.1%) followed by the viability of the fetus (27.0%) (gestational age 23 weeks).

### Reproductive autonomy scale (RAS) and the attitudes toward abortion as well as morning after pill

**Figure 1:**
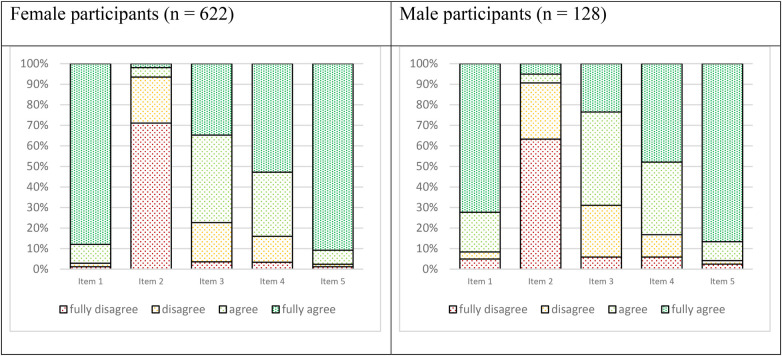
Attitudes toward abortion among university students (*n* = 750). Distribution of responses to five statements regarding abortion attitudes, shown separately for female and male participants. Responses were measured on a four-point Likert scale (fully agree, agree, disagree, fully disagree). Values represent percentages of participants per response category. Item 1: “Abortions are a difficult, individual decision; every woman has the right to decide over her own body.” Item 2: “A pregnancy is the natural consequence of sexual intercourse, and this natural process should not be interfered with.” Item 3: “In the case of a severely impaired child, a couple should have the right to an abortion regardless of the stage of pregnancy.” Item 4: “Minors should also be able to access free and unbureaucratic abortions for social reasons.” Item 5: “After a rape (criminological indication), an abortion should be free of charge and easy to obtain”.

Regarding attitudes toward abortion, 87.9% of women fully agreed with the statement that a woman should have the right to make decisions about her own body, compared to only 72.3% of men (*p* < 0.001). The majority of participants disagreed with the statement that pregnancy is the natural consequence of sexual intercourse and that this natural process should not be interfered with (women: 71.1%, men: 63.9%, *p* = 0.12). Most participants also fully agreed that abortion should be free of charge and easily accessible after a rape (women: 90.8%, men: 86.6%, *p* = 0.48). While the majority of the sample generally agreed with the statements that couples should have the right to terminate a pregnancy at any stage in the case of a severely impaired child, and that minors should have access to free and unbureaucratic abortions for social reasons, only about one-third fully agreed with the former statement and half with the latter. For details, see Figure 1.

Within the female population, greater reproductive autonomy was globally associated with a more liberal stance towards abortion (RAS total score: *p* = <.001). Women with higher freedom of coercion were also more likely to state that every woman has the right to decide for her own body (*r* = 0.10, *p* = 0.025) while they opposed the statement that pregnancy is a natural outcome of sexual activity and should not be interfered with (*r* = −0.10, *p* = 0.027).

Women with a more autonomous form of reproductive decision making also opposed the notion that pregnancy is a natural outcome of sexual activity and should not be interfered with (*r* = −0.12, *p* = 0.008) while agreeing to the statement that abortion should be legal regardless of the gestational age if the foetus was severely disabled (*r* = 0.12, *p* = 0.007) and that minors should get access to free and unbureaucratic abortions based on social indication (*r* = .12, *p* = 0.008). A similar response pattern was observed for women with a more autonomous form of communication, who agreed with all statements (*p* = 0.023–<.001). For details, see Supplementary Table S5 (Supplementary).

### Religious beliefs, field of study and attitudes toward abortion and the morning-after pill

No differences were found on the following statements depending on the participants' religious beliefs: “Abortions are a difficult, individual decision; every woman has the right to decide over her own body” (*p* = 0.60), “In the case of a severely impaired child, a couple should have the right to an abortion regardless of the stage of pregnancy” (*p* = 0.58), and “After a rape (criminological indication), an abortion should be free of charge and easy to obtain” (*p* = 0.94).

However, we found differences in some statements depending on the confession: self-identified Christians were significantly less likely to fully disagree with the statement “A pregnancy is the natural consequence of sexual intercourse, and this natural process should not be interfered with” (65.3% vs. 80%–100%, *p* = 0.006) compared to Muslims/participants without confession. Conversely, self-identified Muslims were significantly more likely to fully disagree with the statement “Minors should also be able to access free and unbureaucratic abortions for social reasons” (20.0% vs. 0%–5%, *p* = 0.005) compared to Christians/ participants without confession.

The use of the morning-after pill was comparable across most religious groups (33.3%–44.2%), with the exception of Buddhists, all of whom reported using it (100.0%). Of note, only 5 participants assigned themselves as Buddhists.

The participants' field of study was not significantly associated with attitudes toward abortion (*p* = 0.13–0.96) or with the use of the morning-after pill (*p* = 0.65).

## Discussion

This study provides novel insights into abortion-related knowledge, attitudes, and reproductive autonomy among Austrian university students. While participants generally expressed supportive views toward reproductive self-determination, factual knowledge regarding abortion laws and available services was limited. Only about half of the respondents knew that abortion is legally regulated in Austria, and many were unaware of where services are offered or what they cost. These findings are consistent with international studies indicating that liberal legal frameworks do not necessarily translate into public awareness or knowledge about access pathways (13). In Austria, abortion is decriminalized (exempt from punishment) within the first three months from the beginning of pregnancy under the so-called “Fristenregelung” (§ 97 StGB). While the procedure remains technically embedded within the Criminal Code, it is not punishable during this period following a prior medical consultation. Nonetheless, the procedure must be paid for privately, which, together with the concentration of services in urban areas, creates significant access barriers. These barriers were poorly understood by participants, highlighting the need for clearer, more accessible information, particularly for young adults and students outside the medical field.

These findings align with European and international research showing limited public knowledge with access pathways even where early abortion is permitted (16). Out-of-pocket payment and the concentration of providers in urban centers are well-described access barriers- a situation which also applies to the Austrian setting (17, 18).

Medical students in our sample were more likely than their peers to identify the legal framework and service-related aspects correctly. This aligns with findings from UK-based studies (19, 20) showing that comprehensive abortion education improves both knowledge and professional confidence. Importantly, abortion-related teaching during undergraduate education, not just during clinical training, has been associated with greater willingness to provide abortion care in future (21). Still, curricular gaps remain evident: a substantial proportion of medical students in international studies reported receiving little or no formal training on abortion, and many expressed a desire for more, particularly in the area of communication with patients (20). Our findings support the argument that systematic inclusion of abortion-related content in health professions curricula is both necessary and desired.

The data indicate that the issue of abortion is neglected in both European and non-European countries, regardless of legislation or religious background. Given that the demand remains constant, improved training for medical students must be implemented.

In non- medical students, knowledge about legal frameworks and services of abortion was even more limited. In a survey among nursing students in Thailand, more than half of the students did not even know that the abortion law had been changed one year ago (22). This is in line with results of a systematic review which showed that more than half of the women were not aware of the legal regulations in their country (23). No correlation between knowledge and liberal legislation was found.

Female students felt less informed than male students about abortion law and more likely to perceive abortion as a social taboo. Women opted more often for more information about abortion and a compulsory abortion course in the medical curriculum This gendered perception of stigma reflects broader findings in literature, where abortion stigma is shown to affect both care seekers and providers, contributing to secrecy, emotional distress, and poorer care experiences (24). Women's heightened sensitivity to stigma in our sample may reflect a greater personal relevance of the issue and a stronger anticipation of social sanctioning. Importantly, the persistence of stigma even in liberal legal environments suggests that improving knowledge alone is insufficient. Cultural narratives and public discourse must also shift to reducing stigma and normalize abortion as part of routine reproductive healthcare.

A consistent pattern emerged linking higher reproductive autonomy with more liberal attitudes toward abortion, particularly among female students. These findings echo previous research demonstrating that reproductive autonomy is associated with contraceptive use and support for reproductive rights. Our study extends these associations to an Austrian student population and shows that reproductive autonomy is not only relevant in contexts of restrictive legislation, but also in liberal democracies where access is formally protected. Interestingly, we did not observe significant differences in attitudes or knowledge by gender, religious affiliation, or rural vs. urban origin—contrary to prior research from other contexts (12). This may be explained by the relatively homogenous, predominantly liberal Christian background of our study population. Still, religious affiliation was differentially associated with specific items: for example, Christian participants were less likely to strongly reject the idea that pregnancy should not be interfered with, and Muslim participants more often disagreed with abortion access for minors based on social grounds. At the same time, the reported use of the morning-after pill was high and relatively uniform across religious groups, suggesting that behavioral practices may not fully align with stated beliefs. We interpret the results regarding religion with great caution, as it is also unclear whether religion is actively practiced or was assigned at birth. Here, we would like to limit ourselves to a straightforward description of our findings and refrain from drawing any general conclusions from them.

Overall, lack of knowledge of sources of care as well as legal regulations may lead to a delay of treatment and even to more fear due to stigma, especially in adolescent girls (25). Regarding the legal context of consent, it is important to note that in Austria, minors from the age of 14 are generally considered capable of providing independent informed consent for medical procedures, including abortion, provided they possess the necessary maturity and capacity for judgment (Einsichtsfähigkeit). This legal framework aims to support reproductive autonomy, yet our findings suggest that students perceive the need for even more “unbureaucratic” access for this vulnerable group. Lack of knowledge regarding these legal rights may further contribute to delays in seeking care and increased fear due to social stigma, especially among adolescents. The fact that abortion services are not covered by public health insurance and must be paid for privately creates a significant socioeconomic barrier that disproportionately affects young adults and those in education, regardless of the formal legal decriminalization. Therefore, improved information campaigns and a more open discourse on this topic are required for the well-being of women, even if the respective legislation may not support such efforts.

We observed strong endorsement of bodily autonomy, especially among women, and a consistent pattern whereby higher reproductive autonomy (total and subscales) correlated with more liberal attitudes. Prior work links greater reproductive autonomy to contraceptive use (10, 26) and to supportive views of reproductive rights; our study extends these associations to an Austrian student population and to the topic of abortion.

The observed link between reproductive autonomy and attitudes towards abortion raises important questions about the relationship between legal frameworks and individual agencies. It remains unclear whether high reproductive autonomy fosters liberal legislation, or whether liberal laws enable greater autonomy. Under restrictive legal conditions, greater individual autonomy may be necessary to develop or maintain pro-choice views, whereas in liberal settings, such attitudes may be more widespread and less consciously developed. The extent to which legal environments shape individual autonomy, or are themselves shaped by it, deserves further investigation. Existing research from Ghana (27), for instance, shows that women with high reproductive autonomy were more likely to access abortion services and to report a history of abortion. If reproductive autonomy is indeed a prerequisite for accessing abortion services, then legislation alone is insufficient to ensure equitable access.

### Strengths and limitations

This study has several strengths, including a large sample size and the use of a validated measure of reproductive autonomy. However, the sample consisted mainly of highly educated, predominantly female students of similar age, which may limit the generalizability of the findings.

Furthermore, the use of convenience sampling and the inability to determine an exact response rate limit the representativeness of the sample and introduce potential selection bias. Participants who chose to respond may differ systematically from those who did not, which may affect the generalizability of the results. Additionally, the sample may overrepresent students from health-related disciplines, who are likely to have higher baseline knowledge and potentially more favorable attitudes toward the topic, thereby inflating estimates of knowledge and reducing observed levels of stigma.

The sample includes students from diverse disciplines but may not fully represent the broader Austrian student population. Furthermore, eligibility with respect to being based in Tyrol relied on self-report and enrolment at a Tyrolean university and was not externally verified (e.g., through official residence records).

The reliance on self-reported data introduces the possibility of social desirability bias, as participants may have provided responses they perceived as socially acceptable rather than reflecting their true beliefs or knowledge. This may have led to an underestimation of stigmatizing attitudes and an overestimation of knowledge levels.

In addition, as multiple statistical tests were conducted without adjustment for multiple comparisons, there is an increased risk of type I error, and findings should be interpreted as exploratory.

Also, men and trans* individuals could not be included due to limitations in the current version of the Reproductive Autonomy Scale (15). Gender-diverse participants (*n* = 6) were excluded from the analyses due to small subgroup size. While this decision was made to ensure statistical validity, it limits the inclusivity of the study and restricts the ability to draw conclusions about gender-diverse populations. Their exclusion may also obscure important variations in knowledge and stigma perceptions across gender identities.

Taken together, these factors may affect the external validity of the study and should be considered when interpreting the results. Future research should aim to

Future research should focus on adapting such tools for more inclusive use and examining how autonomy and abortion attitudes manifest across a broader range of gender identities and social contexts and also incorporate methods to reduce social desirability bias.

### Implications for medical education, practice, and policy

To translate these findings into practice, we propose specific actions: To further enhance equitable access, we suggest addressing the significant financial barrier identified, social funds or student-specific subsidy programs should be established to cover the costs of abortion procedures for those in financial need, as out-of-pocket payments remain a major hurdle. Transparent and accessible information on abortion services, including costs and locations, is essential. In Austria, the lack of statutory coverage and uneven service distribution hinder access, particularly for minors or those facing social vulnerability. Providing accurate, multilingual resources through public and university health services could help close this informational gap and promote equitable access to abortion care.

Second, the integration of interdisciplinary seminars on reproductive rights into general university curricula. Such programs would not only enhance health literacy among non-medical students but also actively contribute to reducing the perceived social taboo by fostering an evidence-based public discourse on reproductive autonomy.

Furthermore, efforts to reduce abortion stigma must be prioritized, as stigma remains a key barrier to both seeking and providing care. Normalizing abortion as a part of routine sexual and reproductive healthcare, independent of legal status, can help mitigate these effects.

## Conclusions

Among this sample of Austrian university students, support for reproductive autonomy is high, but profound knowledge about the legal framework, providers, and costs remains limited, particularly outside medical professions. Female students report greater awareness of legal timelines but also greater perception of social taboo; higher reproductive autonomy is linked to more liberal attitudes. Educational reforms, stigma-reduction strategies, and clearer access information are feasible levers to improve reproductive health literacy and equity in Austria.

## Data Availability

The raw data supporting the conclusions of this article can be made available on request by the authors, without undue reservation.
