# Supplementary material for: Abortion knowledge, attitudes and reproductive autonomy among university students in Austria: a cross-sectional study
Source: Front Reprod Health. 2026 May 22;8:1820413. doi: 10.3389/frph.2026.1820413 (PMC13236956; doi:10.3389/frph.2026.1820413)
Supplement: Supplementary file 1 [file Table1.docx]

**e- Table 1: Summary of the Study Flow (Recruitment and Selection)**

| **Phase** | **Description** | **Count (n)** |  |
| --- | --- | --- | --- |
| **Recruitment** | Students who accessed the survey via web link or QR code | *>1200* |  |
| **Eligibility** | Students based in Tyrol who provided informed consent | 937 |  |
| **Data Cleaning** | Participants with complete sociodemographic data and Reproductive Autonomy Scale (RAS) scores | **756** |  |
| **Exclusion** | Gender-diverse individuals (due to small sample size for analysis and RAS scale limitations) | **6** |  |
| **Final Sample** | **Total number of students included in the final analysis** | **750** |  |
|  | – Female participants | **622** |  |
|  | – Male participants | **128** |  |

e-Table 5. Correlations between the Reproductive Autonomy Scale (RAS) and the Attitudes towards Abortion

|  | RAS – total score | | RAS – Freedom of Coercion | | RAS – Communication | | RAS – Decision making | |
| --- | --- | --- | --- | --- | --- | --- | --- | --- |
|  | r | p | r | p | r | p | r | p |
| Abortion is a difficult, individual decision; every woman has the right to decide what happens to her body. | .16 | <.001 | .10 | .025 | .06 | .20 | .18 | <.001 |
| Pregnancy is the natural consequence of sexual intercourse, and this natural process should not be interfered with. | -.20 | <.001 | -.10 | .027 | -.12 | .005 | -.22 | <.001 |
| In the case of a severely disabled child, a couple should have the right to terminate the pregnancy regardless of the stage of pregnancy | .16 | <.001 | -.02 | .62 | .12 | .007 | .097 | .023 |
| Minors should also be able to have an abortion free of charge and without bureaucracy for social reasons. | .17 | <.001 | -.03 | .49 | .12 | .006 | .21 | <.001 |
| Following rape (criminal indication), abortion should be available free of charge and without bureaucratic hurdles. | .08 | .06 | .006 | .90 | .033 | .45 | .12 | .004 |
| In your opinion, to what extent is the topic of abortion a social taboo? | .08 | .052 | -.07 | .12 | .10 | .03 | .08 | .08 |
